# The Effect of Magnetic Field on Catalytic Properties in Core-Shell Type Particles

**DOI:** 10.3389/fchem.2020.00163

**Published:** 2020-03-12

**Authors:** Emma Westsson, Stephen Picken, Ger Koper

**Affiliations:** Department of Chemical Engineering, Delft University of Technology, Delft, Netherlands

**Keywords:** magneto-electrocatalysis, fuel cell, electrochemistry, oxygen reduction (ORR), magnetic field—effects

## Abstract

Magnetic field effects can provide a handle on steering chemical reactions and manipulating yields. The presence of a magnetic field can influence the energy levels of the active species by interacting with their spin states. Here we demonstrate the effect of a magnetic field on the electrocatalytic processes taking place on platinum-based nanoparticles in fuel cell conditions. We have identified a shift in the potentials representing hydrogen adsorption and desorption, present in all measurements recorded in the presence of a magnetic field. We argue that the changes in electrochemical behavior are a result of the interactions between the magnetic field and the unpaired spin states of hydrogen.

**Graphical Abstract:**
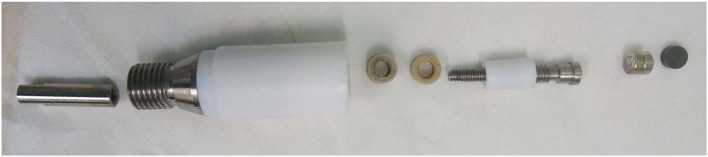
By modifying a rotating disc electrode, permanent magnets could be positioned very close to the electrode surface.

## Introduction

Our aim is to explore how the catalytic behavior of a specific electrochemical process can be altered, and hence, better understood. We are here considering magnetic fields as a handle to influence electrochemical processes. Subatomic particles, like electrons, have mass, spin, and charge. It is fair to assume that at least one of these properties—spin—can be perturbed by the presence of a magnetic field. Spin is intrinsic and gives rise to a magnetic moment that can be manipulated.

Steiner et al. were the first to compose a bird's-eye review on magnetic effect on chemical reactions, including examples like magnetic fluorescence quenching, photo-addition of SO_2_ to pentane, thermal decomposition of organic peroxides, reactions of alkali-metal alkyls with alkyl halides etc. (Steiner and Ulrich, 1989). Furthermore, the review refers to a variety of reports on magnetic field effects on photophysical phenomena in (organic) molecular crystals, such as luminescence and photoconductivity. Even examples of simple experiments taking place between two laboratory magnets in which the reaction rate between organic radicals were measured proved to show a remarkable increase in the reaction rate (Turro and Kraeutler, 1980). As described by Steiner et al. most magnetic field effects in chemical processes take place in liquid solutions, mostly as a result of radical pair mechanism (Okazaki and Shiga, 1986; Steiner and Ulrich, 1989). In gas phase reactions on the other hand, the radical geminate re-encounter is unlikely and in solid phase reactions radical pairs do not separate easily. Shovkovy et al. presented a review of a theoretical approach to magnetic field effects on chemical reactions (Shovkovy, 2013). Torun et al. describe how the existence of a local magnetic moment of RuO_2_ catalyst surfaces conserves the angular momentum and enables the production of magnetic oxygen from non-magnetic water (Torun et al., 2013).

For electron transfer reactions reports are scarcer. One of the first examples were presented by Periasamy et al. studying the electron-transfer reaction between diazabicyclooctane (DABCO) and fluorenone triplet in propylene carbonate (Periasamy and Lindschitz, 1979). For magnetic field effects in electrocatalysis there are even less reports. Leddy and co-workers were to our knowledge the first group working on magnetically modified electrodes with the purpose of enhancing electron transfer kinetics. They studied the magnetic field effect on Hydrogen Evolution Reaction (HER) on non-catalytic surfaces. Furthermore, they studied oxidation of CO_2_ on a magnetically modified platinum electrode. The electrodes were made with magnetic micro-particles attached to the electrode surface, so that it could sustain a permanent magnetic field. Their studies show that oxidation of carbon monoxide at such modified Pt electrode surfaces are considerably altered compared to electrodes without magnetic micro-particles, through spin polarization (Dunwoody et al., 2005). The oxidation of carbon monoxide occurred at 600 mV lower overpotential. The enhancement of the electron transfer rate is claimed to originate from the suppression of entropy of the electron spin and hence a lowered activation barrier.

Jonsson et al. studied the effect of magnetic states on the reactivity of an iron surface using Density Functional Theory (DFT) calculations. Their results suggest that the charge-transfer between the catalyst surface and the adsorbate is strongly affected by spin-structure. In their study H_2_ and CO adsorption and dissociation was modified by changes in spin-structure (Melander et al., 2014).

Recently, Galán-Mascarós et al. showed that highly magnetic electrocatalysts like mixed Ni-Fe-Zn-based oxides exhibit higher activities for Oxygen Evolution reaction (OER) upon applying a magnetic field to the anode (Garcés-Pineda et al., 2019). Also a study by Peng et al. on the magnetic field effects on a Co-oxide electrocatalyst confirms that the OER can be improved by placing the electrolysis cell in between permanent magnets with a moderate field (Li et al., 2019). They further add the effect of the directionality of the magnetic field on the overpotential and Tafel slope. The same material was further investigated by Wei et al. for possible improvements in the catalytic activity toward Oxygen Reduction Reaction (ORR) (Zeng et al., 2018). A small improvement in the selectivity toward the 4-electron pathway is achieved by applying an external magnetic field. These recent studies show the effect of spin-polarization by a magnetic field on the catalytic properties of transition metal oxides.

In this paper we present our study on the effects of an external magnetic field on the electrocatalytic processes taking place on four platinum-based electrocatalysts. To our knowledge, this is the first study of the interactions of Pt and hydrogen under an externally applied magnetic field. For this purpose, we have integrated strong magnets into the shaft of a Rotating-Disc Electrode and record the electrochemical processes on Pt in Ar- or O_2_ saturated acid electrolyte, in the presence and absence of a magnetic field. Due to the unpaired spin states in hydrogen as well as in oxygen, these species respond to a certain extent to a magnetic field.

### Hydrogen Adsorption and Desorption

**Figure 1:**
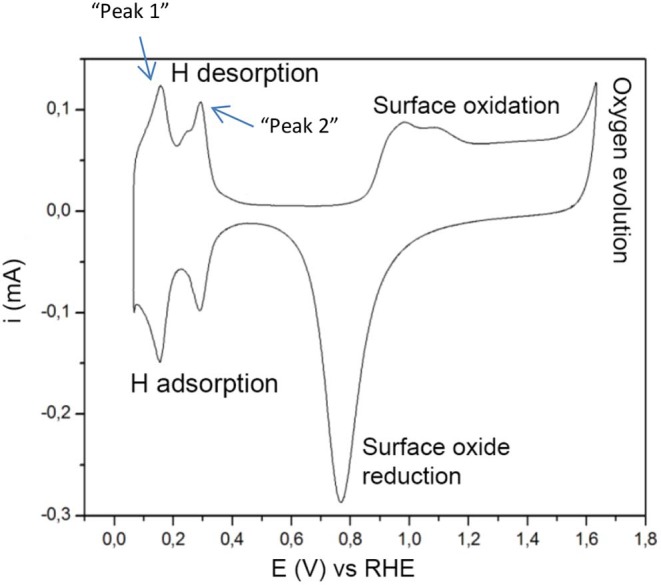
The typical features of cyclic voltammetry on Pt catalysts in acidic electrolyte. The hydrogen desorption peaks are further referred to as “peak 1” and “peak 2.” The figure is based on concepts from Łukaszewski et al. (2016).

The main electrochemical processes taking place in these conditions are firstly—if oxygen is present—Oxygen Evolution Reaction (OER) and Oxygen Reduction Reaction (ORR) with a thermodynamic equilibrium potential of 1.23 V vs. SHE, see Figure 1. Oxygen adsorption and desorption proceeds these reactions. The ORR and OER are more thoroughly described in (Norskov, 2000; Koper, 2008; Zhang, 2008; Diaz-Morales et al., 2018). The region in between the surface oxide formation/reduction region and the hydrogen adsorption/desorption region is usually referred to as the “double layer region.” In this region no faradaic processes take place but only capacitive processes (Łukaszewski et al., 2016). Secondly, the Hydrogen Evolution Reaction (HER) and the Hydrogen Oxidation Reaction (HOR) have a thermodynamic equilibrium potential of 0 V vs. SHE and characterize the features in the cyclic voltammogram around 0 V. When the potential is kept >0 V (vs. SHE) only Hydrogen adsorption and desorption takes place, often referred to as H under-potential deposition (H_upd_).

Adsorption can be sorted as: (1) molecularly chemisorbed, (2) atomically chemisorbed and (3) molecularly physisorbed (Oudenhuijzen et al., 2001; Roduner, 2014; Kulkarni et al., 2018). Molecular physisorption refer to electrostatic interactions between Pt and molecular H_2_, where no electrons are shared and no dissociation occurs. As described elsewhere (Oudenhuijzen et al., 2001) molecularly chemisorbed H_2_ is highly unlikely. H_2_ will immediately dissociate implying that chemisorption of H_2_ on Pt is atomic to the largest part.

The mechanism of hydrogen adsorption and evolution on platinum has been extensively studied (Kreuer, 2013; Zheng et al., 2014; Murthy et al., 2018). This process is fast and electrochemically reversible and the equilibrium surface coverage depends on the electrode potential. Cyclic voltammograms recorded for platinum in acidic electrolyte show distinctive peaks at 0–0.4 V vs. RHE. There seems to be a general consensus concerning the origin of the most prominent peaks (Oudenhuijzen et al., 2001; Kreuer, 2013; Sarkar et al., 2013; Łukaszewski et al., 2016; Diaz-Morales et al., 2018), located around 0.125 and 0.27 V, representing the (110) and (100) step sites on Pt, in the following manner described in detail by Diaz-Morales et al. (2018):

$$\begin{array}{l} {\text{H}}^{+}+{\text{e}}^{-}{+}^{*}\text{hkl}⇄\text{Hads},\text{hkl} \end{array}$$

where ^*^hkl indicates a free site on the Pt surface with hkl Miller indices.

## Materials and Methods

For this study three electrocatalysts were made according to our bi-continuous micro-emulsion based core-shell synthesis described in Westsson and Koper (2014) and Westsson et al. (2019): Pt@Fe nanoparticles, Pt@Cu nanoparticles, and pure Pt nanoparticles supported on carbon made in bi-continuous micro-emulsion. Further, commercial Pt on carbon (60 wt% Pt on Vulcan XC-72R, Johnson Matthey, UK) was used (from now on denoted “pure Pt”).

### Setup

**Figure 2:**
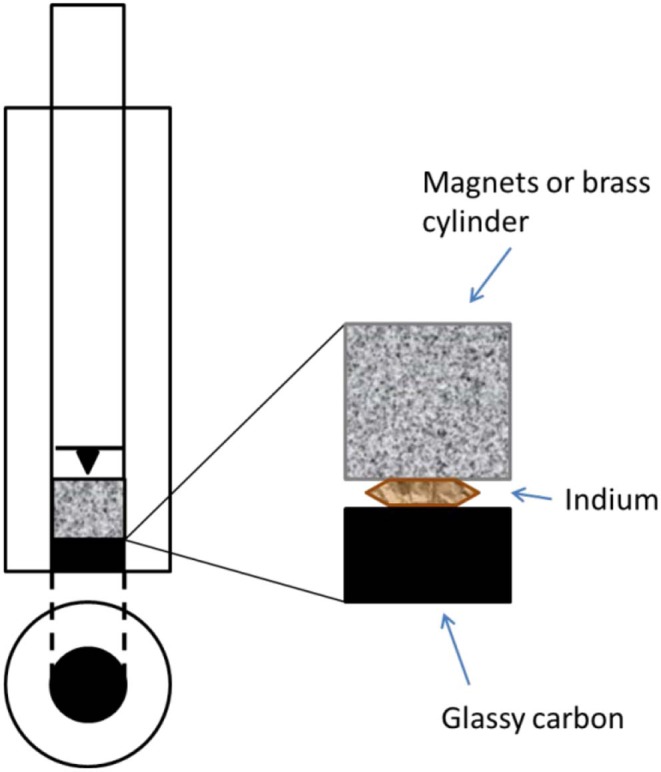
A schematic picture of the modified RDE shaft.

To be able to achieve a magnetic field, as strong and as close as possible to the catalyst layer, the rotating disk electrode was modified and perfected in many steps. A Pine Instrument RDE electrode with removable glassy carbon disk of 5 mm was used as starting material for the build-up of an electrode with the option to be both magnetic and non-magnetic. To accommodate the magnets right in between the glassy carbon disk and the spring-loaded shaft, both the disk and the shaft had to be reduced in size. The length of the glassy carbon disk was reduced from 5 mm to 2 mm by ultra-fine polishing, thanks to *Surface Preparation Laboratory*, Zaandam, NL. A new, shorter shaft was made, keeping the spring-loaded tip to ensure good electrical contact with the magnets. The “cavity” inside of the modified RDE electrode was made such that it could accommodate either only magnets, only a non-magnetic brass cylinder or half-half magnets/brass as a crude way of varying the magnetic field strength. See Figure 2 for a schematic illustration.

The magnets (*Supermagnets*, Dresden, Germany) are Neodymium magnets of size 5 mm in diameter and 2, 3, and 5 mm long cylinders, coated with nickel. The magnets are of grade N52 which correspond to a magnetic field strength of ~0.4 T in close vicinity to the catalyst layer.

### Experimental

The catalyst samples, Pt@Fe, Pt@Cu, and pure Pt (from now on denoted “Pt micro-emulsion”), were prepared and characterized according to our micro-emulsion synthesis of core-shell particles described in Westsson and Koper (2014) and Westsson et al. (2019), in which a 3 nm metal core is firstly synthesized inside the aqueous channels of a dense micro-emulsion. The shell is subsequently added through galvanic replacement of surface atoms of the core in favor of the shell metal. In a third step the carbon support (Vulcan XC-72R) is added and the supported core-shell particles are washed in a fourth step. In order to get the most accurate comparison between activity measurements made with the magnetic electrode configuration relative to the non-magnetic configuration, the measuring sequence proved to be crucial. Two consecutive measurements—with and without magnetic field—had to be made either on two different ink layers and running the risk of having differences between the layers, or on one single layer in which both measurements with magnets and brass cylinder were done on the exact same layer. In the latter case the risk is that the layer gets slightly damaged upon switching the interior of the electrode, since it involves moving the glassy carbon disk. Furthermore, there is a risk that the potential cycling permanently changes the catalyst through the sets of measurements, in other words, a “memory effect.” A needle is used to push the catalyst coated glassy carbon disk into the PTFE holder upon removal/insertion of magnets into the shaft. “m” represents magnetic configuration and “b” represents brass cylinder, i.e., non-magnetic configuration in the figures. Table 1 explains how the measurements were labeled according to magnetic configuration and order.

**Table 1:** Illustrates an example of how the measurements were executed and how they were labeled accordingly.

	**RDE configuration**	**Label**	**Pt@Fe**	**Pt@Cu**	**Pure Pt**	**Pt micro-emulsion**
1st catalyst layer	1. Magnetic	m1	Cyclic voltammetry and hydrodynamic measurements			
	2. Non-magnetic	b2				
	3. Magnetic	m3				
2nd catalyst layer	1. Non-magnetic	b1	Cyclic voltammetry and hydrodynamic measurements			
	2. Magnetic	m2				
	3. Non-magnetic	b3				
3rd catalyst layer etc.						

An optical microscope was used to estimate the loss of catalyst from the glassy carbon electrode due to re-insertion of the glassy carbon into the RDE tip. A micrometer microscopy ruler was used to measure the length and width of the scratches and a percentage of the total electrode area could be estimated, assuming a homogeneously deposited catalyst layer.

The catalytic activity measurements were carried out under acidic conditions according to a standardized RDE procedure (Garsany et al., 2014). The electrolyte used was a 0.1 M HClO_4_ solution. The reference electrode was a RHE electrode—in essence a Pt wire with freshly prepared H_2_ gas—and the counter electrode was a platinum wire winded into a spiral shape. For all electrochemical measurements, an Autolab PGSTAT 20 potentiostat was used, along with a 3-electrode cell and a Rotating Disc Electrode (RDE) from Pine Instruments with a 5 mm in diameter glassy carbon disk and hence an electrode area of 0.198 cm^2^. The working electrode were prepared by thoroughly polishing the glassy carbon disk with 1.0, 0.3, and 0.05 μm alumina particle polishing suspensions, rinsing in between each step. Any residual polishing medium was cleaned off in an ultrasonic bath. An ink was made by mixing 6.0 mg of catalyst powder (i.e., carbon + core-shell particles) with 4.56 μl Nafion suspension (5 wt%) and 12 ml isopropanol. The ink was mixed using an ultrasonic bath. To make the catalyst ink layers on the electrode 13 μl of catalyst ink was drop casted on the disk. All experiments were conducted in an electrolyte saturated with Ar for the cyclic voltammetry and O_2_ for the hydrodynamic voltammetry using rotation speeds of 400, 900, 1,600, and 2,500 rpm according to commonly used evaluation procedures, as described in Gasteiger et al. (2005) and Garsany et al. (2014). Cyclic voltammograms recorded in Ar-saturated electrolyte were measured at scan speeds of either 50 mV/s or 100 mV/s. Scan speed for hydrodynamic voltammetry was 5 mV/s. In a first electrochemical cleaning step, potential cycling at a speed of 100 mV/s for 50 cycles was used. The ECSA and ECSA loss was calculated according to commonly used methodology described first by Trasatti (1991). The scan rate used was 50 mV/s.

## Results

### Sample Preparation

**Figure 3:**
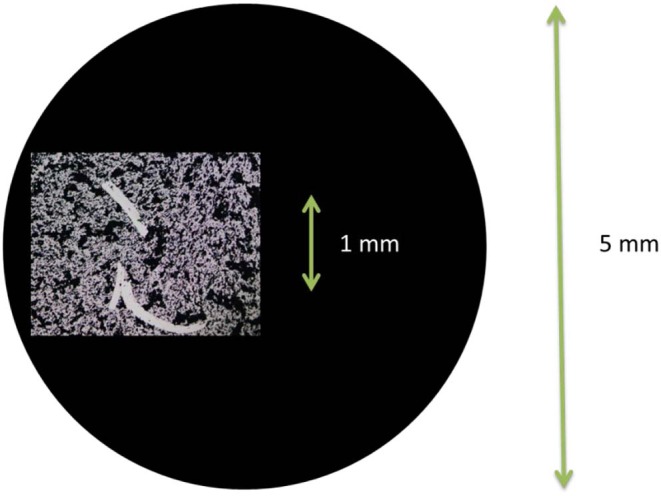
Photo using optical microscope of the scratches made in the catalyst layer when re-fitting the catalyst coated glassy carbon disk into the PTFE holder after switching configuration. The black circle represents the relative size of the glassy carbon disk.

Each time the glassy carbon is being pushed into the PTFE holder by the needle, the geometrical area loss estimated by optical microscopy is ~1–5% each time the configuration is switched, see Figure 3 for an example. This loss is inevitable unless the architecture of the electrode setup is completely altered.

### Cyclic Voltammetry

**Figure 4:**
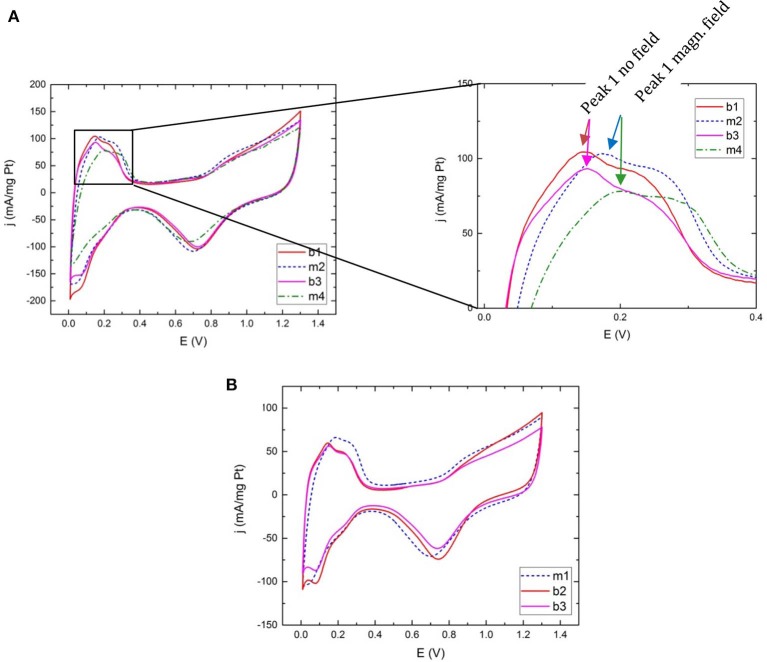
Cyclic voltammograms for Ar-saturated electrolyte for **(A)** commercial Pt at 100 mV/s with close-up on the hydrogen desorption region and **(B)** commercial Pt measured at 50 mV/s. Peak 1 corresponds to the desorption of hydrogen from Pt (110) sites and peak 2 represents desorption from Pt (100). The legend refers to the measurement sequence (1–4) and magnetic (m) or non-magnetic (b) measurement configuration as explained in Table 1.

In the cyclic voltammograms in Figure 4A loss in catalyst surface area, estimated by hydrogen adsorption and desorption peak areas, is observed upon switching configuration. This is, at least partially, due to the small damage to the catalyst layer arising from pushing the glassy carbon into the PTFE holder. As an example, in a measurement of the sample “Pt@Fe” (Figure S1) the ECSA amounts to: m1 = 70 m^2^/g, b2 = 62 m^2^/g, and m3 = 53 m^2^/g. The calculation of specific activity is based on these values. The loss estimated by ECSA is larger than the area loss estimated with optical microscopy.

Cyclic voltammograms for Pt@Cu, Pt@Fe, and pure Pt made in bi-continuous micro-emulsion are presented in Figures S1, S2 the only prominent result being the decrease in current density upon switching between the two configurations. The plots illustrate similar peak features in magnetic as well as non-magnetic configuration.

On the contrary, in cyclic voltammograms using commercial pure Pt as catalyst the hydrogen adsorption and desorption peaks are more prominent since the particles are better dispersed on the carbon support. Not only are they higher in relative terms but a behavioral difference between magnetic configuration, “m” and non-magnetic configuration, “b” (for brass) is unfolding. Although the number of different ink layers analyzed with a sequence of magnetic and non-magnetic measurements are limited to ~10, each and every voltammogram contribute to a pattern of peaks shifting to higher potentials when scanning toward more oxidizing potentials, and vice versa, when a magnetic field is present, independent of measurement sequence. Figure 4 display voltammograms for samples measured at both 100 mV/s scan speed and 50 mV/s, using different measurement sequences. Figure 4A highlights the significant hydrogen desorption peak position difference between measurements done with and without the presence of a magnetic field. In Figure 4B the measurement order is magnetic-nonmagnetic-nonmagnetic in order to rule out any contribution from the configuration switch itself. Peak positions remain the same between “b2” and “b3.” A small shift in the position of oxygen desorption is visible in some measurements. In this initial study the focus has however been on the hydrogen region.

### Hydrodynamic Voltammetry

After each measurement in Ar-saturated electrolyte, hydrodynamic voltammetry in O_2_-saturated electrolyte was carried out analyzing the mass activity and specific activity of each configuration for each catalyst layer.

**Figure 5:**
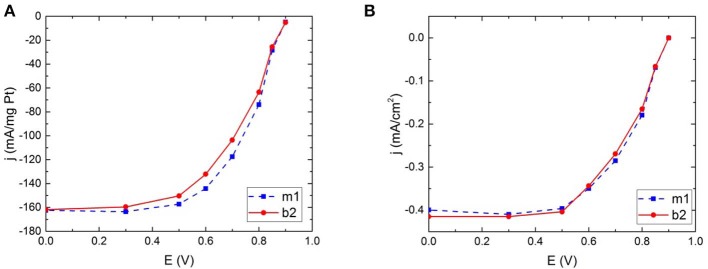
Hydrodynamic voltammograms for commercial Pt illustrating the catalytic activity for oxygen reduction in the two different electrode configurations; magnetic field and no magnetic field, calculated both as **(A)** mass activity and **(B)** specific activity.

An obvious and expected effect of the loss of catalyst is reflected in the loss of mass activity between the measurements, illustrated in Figure 5 as an example and in SI (Figure S5). If the total electrochemically active surface area of the layer is taken into account the measurements—magnetic and non-magnetic—overlap to a great extent. As results suggest, no significant difference in catalytic activity was detected between magnetic and non-magnetic measurements.

## Discussion

The largest contribution to the errors in the measurements is the contact resistance upon switching between magnetic and non-magnetic configuration for a single layer. It is however, crucial to perform both types of measurements on a single layer since the influence of the fabrication and permanent change to the catalyst during measurements cannot be ignored.

The loss of ECSA in percent between measurements is disproportionally large compared to the observed catalyst layer loss observed by microscope. However, ECSA loss throughout a sequence of measurements does not vary significantly between catalyst layers. As discussed in Westsson et al. (2019) it is problematic to use ECSA as an estimate for surface area on non-standardized surfaces. It can however, serve a purpose as to estimate—within a single catalyst—the relative surface area. The catalyst loss calculated using ECSA is possibly more reliable than the loss estimated by microscopy, since specific activity plots seem to overlap.

Commercial Pt has the highest ECSA which means any effect would be most visible in this sample. Although the peak positions in the other samples do not display a clear shift, they are not countering the result from the commercial platinum. As an attempt to quantify the peak shift in commercial Pt samples, peak deconvolution using two Gaussian curves fitted to the data was carried out, see Figure S6 for an example. Since the hydrogen desorption peaks provided more prominent peaks, they compose the basis for such analysis. Although the adsorption peaks seemingly follow the same trend, a statistical assessment has not been made using that set of data.

**Figure 6:**
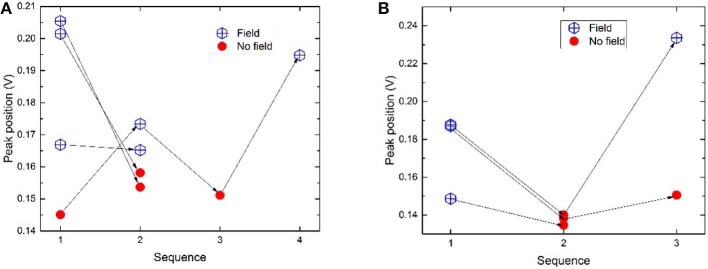
**(A)** Peak position as a function of the measurement order for hydrogen desorption peak 1 for commercial Pt samples measured at 100 mV/s. The arrows connect the measurements done on one same layer. **(B)** Peak 1 positions for samples measured at 50 mV/s. For peak positions of peak 2, see Figure S3.

The positions of the two peaks as a function of measurement order are illustrated in Figure 6. In general peak positions are shifted to higher potentials when a magnetic field is present. When the configuration is “switched” from magnetic to magnetic (i.e., glassy carbon including the catalyst layer was removed and refitted again without changing the magnets for the non-magnetic cylinder) or non-magnetic to non-magnetic the peak positions do not show as much of a peak shift. Such measurements give an idea of the error in terms of peak positions in absence and presence of magnetic field *within one catalyst layer*. These data points are however too few to determine a reliable standard error. Another source of error arises from the reproducibility *between different catalyst layers*. This error is expected to be relatively large. As an example, two measurements named m1 (where “1” denotes the sequence number) from different catalyst layers should ideally overlap, but as illustrated in Figure 6, this is not the case. Nevertheless, the relative shift in peak position upon switching configuration is most relevant when compared with a measurement of the *same* layer.

**Figure 7:**
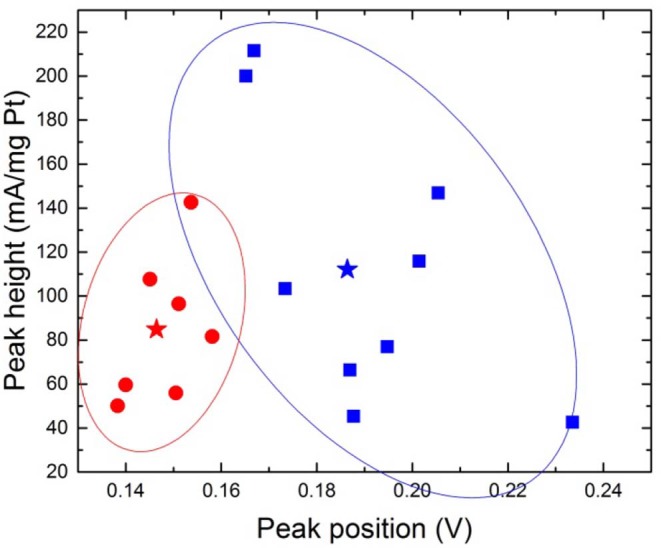
Peak position and peak height for desorption peak 1, where blue square=magnetic configuration and red circle=non-magnetic configuration. The stars represent the means (b: 0.146 ± 0.008; 84.86 ± 33.38, and m: 0.186 ± 0.024; 112.04 ± 62.76). The ovals provide a guide to the eye.

As a summary of all measurements the heights and positions of peak 1 are displayed in Figure 7. Magnetic and non-magnetic measurements clearly form two distinct clouds where the peak positions distinguish the two. The peak heights on the other hand do not separate the two configurations. See Figure S4 for more information.

**Figure 8:**
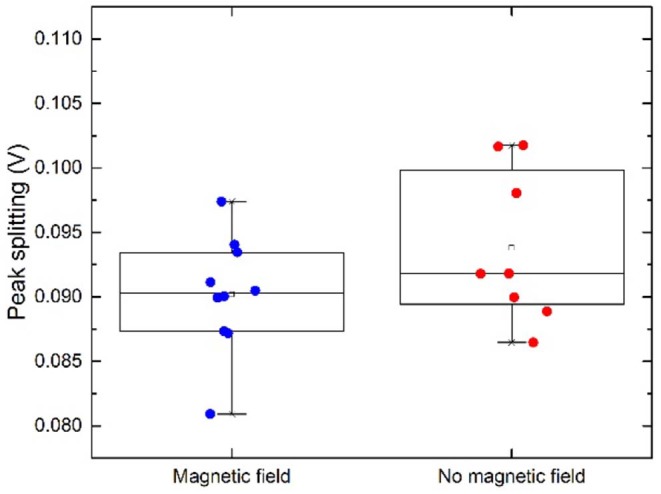
Peak splitting. A small difference between the two configurations is visible but not statistically significant.

When performing a Two Sample *t*-Test where the threshold for statistical significance α = 0.05 is chosen, on the dataset of peak positions (both peak 1 and peak 2) for “m” and “b,” the means and population variances are significantly different between m and b. The average value for peak positions for peak 1 in magnetic configuration is 0.186 V ± 0.024 and 0.146 V ± 0.008 for non-magnetic configuration, illustrated with a star in Figure 7. This leaves a shift of ~0.04 V. The relative distances between the two peaks are presented in Figure 8. The means and variances are not significantly different in a Two Sample *t*-Test. Obviously, due to the small sample population the statistics has limited quality. However, this is an effort to disentangle the effect of the magnetic field on a limited dataset.

The shift in peak positions suggestively caused by the presence of a magnetic field is small but however, presents in all (commercial Pt) samples for various scan speeds. Our limited study on electrocatalytic behavior with and without the presence of a magnetic field suggest that the desorption of H from the Pt surface is slowed down in the presence of a magnetic field.

In these experiments the surface is magnetized or non-magnetized. Any magnetic effect on the potential must be related to the spins of some active species interacting with the field. Hydrogen adsorption and desorption on Pt are two processes in our system. H_2_ itself has two magnetic spins, one of the electron and one of the proton, whereas H^+^ only have spin ± $\frac{1}{2}$. In H_2_ the + 1 and−1 spin state will respond to the magnetic field whereas the antiparallel spin state does not interact with the magnetic field. This implies that 50% of the hydrogen is spin-polarized and 50% is not. Also platinum is magnetisable due to unpaired spins and the magnetic field will influence its band structure (Grechnev, 2009). As an effect, the binding energy of the hydrogen to the platinum surface is changed and that is what is observed in this study. A change in Zeeman energy due to the presence of a magnetic field can affect the activation energy positively or negatively by changing the net enthalpy of the activation barrier and thereby change the redox reaction rate (Ozerovab and Vorobyev, 2007; Zeng et al., 2018). However, if the energy levels in the atoms or molecules are changed by applying a magnetic field through the Zeeman effect the picture might change and the I-V curve peaks no longer represent what would be the case without a magnetic field. An in-depth analysis of the mechanism, however, is beyond the scope of this study. Nevertheless, it is, to our knowledge, the first experimental study discussing the effect of an external magnetic field on hydrogen adsorption on Pt—one of our most important electrocatalysts. Our results point in the same direction as predictions from calculations presented in Melander et al. (2014).

With the current setup and magnetic field strength, a clear and significant effect of magnetic field on the catalytic activity toward oxygen reduction reaction, either onset potential or kinetically limited current, has not been detected or does simply not exist for the catalyst materials studied here.

In conclusion we propose that the changes in the electrochemical behavior observed in this study are due to the interaction of the magnetic states of hydrogen with the catalyst and that this causes a shift in the potentials for the hydrogen adsorption and desorption. At least some states of hydrogen are magnetic—so we presume that the electrochemical processes are influenced by the magnetic states of hydrogen. With the observations from this study in mind, we are stressing the effect of an external magnetic field on both the catalyst and the reactants and the importance of their spin states, previously rarely discussed.

In particular when dealing with catalysis of small symmetric molecules, like H_2_ and O_2_, the activation of these molecules rely on breaking their symmetry. The introduction of an external magnetic field could potentially serve as a handle to control symmetry breaking and hence lower activation barriers. This would provide a modest addition to conventional catalysis approaches.

## Data Availability Statement

All datasets generated for this study are included in the article/Supplementary Material.

## Author Contributions

EW conducted the experiments and wrote the text. GK supervised and contributed with discussions and guidance together with SP.

### Conflict of Interest

The authors declare that the research was conducted in the absence of any commercial or financial relationships that could be construed as a potential conflict of interest.

## References

[B1] Diaz-Morales O. Hersbach T. J. P. Badan C. Garcia A. C. Koper M. T. M. (2018). Hydrogen adsorption on nano-structured platinum electrodes. Faraday Discuss 210, 301–315. 10.1039/c8fd00062j 29987308

[B2] Dunwoody D. C. Ünlü M. Wolf A. K. H. Gellett W. Leddy J. (2005). Magnet incorporated carbon electrodes: methods for construction and demonstration of increased electrochemical flux. Electroanalysis 17, 1487–1494. 10.1002/elan.200503297

[B3] Garcés-Pineda F. A. Blasco M. Castro D. N. López N. Galan-Mascaros J. R. (2019). Direct magnetic enhancement of electrocatalytic water oxidation in alkaline media. Nat. Energy 4, 519–525. 10.1038/s41560-019-0404-4

[B4] Garsany Y. Ge J. St-Pierre J. Rocheleau R. Swider-Lyons K. E. (2014). Analytical procedure for accurate comparison of rotating disk electrode results for the oxygen reduction activity of Pt/C. J. Electrochem. Soc. 161, F628–F640. 10.1149/2.036405jes

[B5] Gasteiger H. A. Kocha S. Sompalli B. Wagner F. T. (2005). Activity benchmarks and requirements for Pt, Pt-alloy, and non-Pt oxygen reduction catalysts for PEMFCs. Appl. Catal. B Environ. 56, 9–35. 10.1016/j.apcatb.2004.06.021

[B6] Grechnev G. E. (2009). Magnetic-field-induced effects in the electronic structure of itinerant d- and f-metal systems. Low Temperature Phys. 35, 638–651. 10.1063/1.3224723

[B7] Koper M. T. M. (2008). Fuel Cell Catalysis: A Surface Science Approach. Hoboken, NJ: Wiley.

[B8] Kreuer K.-D. (2013). Fuel Cells. New York, NY: Springer 10.1007/978-1-4614-5785-5

[B9] Kulkarni A. Siahrostami S. Patel A. Nørskov J. K. (2018). Understanding catalytic activity trends in the oxygen reduction reaction. Chem. Rev. 118, 2302–2312. 10.1021/acs.chemrev.7b00488 29405702

[B10] Li Y. Zhang L. Peng J. Zhang W. Peng K. (2019). Magnetic field enhancing electrocatalysis of Co3O4/NF for oxygen evolution reaction. J. Power Sour. 433:226704 10.1016/j.jpowsour.2019.226704

[B11] Łukaszewski M. Soszko M. Czerwinski A. (2016). Electrochemical methods of real surface area determination of noble metal electrodes – an overview. Int. J. Electrochem. Sci. 11, 4442–4469. 10.20964/2016.06.71

[B12] Melander M. Laasonen K. Jónsson H. (2014). Effect of magnetic states on the reactivity of an FCC(111) iron surface. J. Phys. Chem. C 118, 15863–15873. 10.1021/jp504709d

[B13] Murthy A. P. Madhavan J. Murugan K. (2018). Recent advances in hydrogen evolution reaction catalysts on carbon/carbon-based supports in acid media. J. Power Sour. 398, 9–26. 10.1016/j.jpowsour.2018.07.040

[B14] Norskov B. H. J. K. (2000). Theoretical surface science and catalysis—calculations and concepts. Adv. Catal. 45, 71–129. 10.1016/S0360-0564(02)45013-4

[B15] Okazaki M. Shiga T. (1986). Product yield of magnetic-field-dependent photochemical-reaction modulated by electron-spin-resonance. Nature 323, 240–243. 10.1038/323240a0

[B16] Oudenhuijzen M. K. Bitter J. H. Koningsberger D. C. (2001). The nature of the Pt-H bonding for strongly and weakly bonded hydrogen on platinum A XAFS spectroscopy study of the Pt-H antibonding state shaperesonance and Pt-H EXAFS. J. Phys. Chem. B 105, 4616–4622. 10.1021/jp0108014

[B17] Ozerovab R. P. Vorobyev A. A. (2007). Physics for Chemists. Amsterdam: Elsevier.

[B18] Periasamy N. Lindschitz H. (1979). Cage escape and spin rephasing of triplet ion-radical pairs: temperature-viscosity and magnetic field effects in photoreduction of fluorenone by DABCO. Chem. Phys. Lett. 64, 281–285. 10.1016/0009-2614(79)80513-8

[B19] Roduner E. (2014). Understanding catalysis. Chem. Soc. Rev. 43, 8226–8239. 10.1039/C4CS00210E 25311156

[B20] Sarkar A. Kerr J. B. Cairns E. J. (2013). Electrocatalysis in Fuel Cells - A Non- and Low Platinum Approach. London: Springer 10.1007/978-1-4471-4911-8

[B21] Shovkovy I. A. (2013). “Magnetic catalysis: a review,” in Strongly Interacting Matter in Magnetic Fields, eds Kharzeev D. Landsteiner K. Schmitt A. Yee H.-U. (Berlin: Springer) 13–49. 10.1007/978-3-642-37305-3_2

[B22] Steiner U. E. Ulrich T. (1989). Magnetic field effects in chemical kinetics and related phenomena. Chem. Rev. 89, 51–147. 10.1021/cr00091a003

[B23] Torun E. Fang C. de Wijs G. A. de Groot R. A. (2013). Role of magnetism in catalysis: RuO2(110) surface. J. Phys. Chem. C 117, 6353–6357. 10.1021/jp4020367

[B24] Trasatti P. (1991). Real surface area measurements in electrochemistry. Pure Appl. Chem. 63, 711–734. 10.1351/pac199163050711

[B25] Turro N. J. Kraeutler B. (1980). Magnetic field and magnetic isotope effects in organic photochemical reactions. a novel probe of reaction mechanisms and a method for enrichment of magnetic isotopes. Acc. Chem. Res. 13, 369–377. 10.1021/ar50154a005

[B26] Westsson E. (2019). Low noble metal content catalysts for hydrogen fuel technology. (Ph.D. thesis dissertation). Delft: University of Technology Netherlands.

[B27] Westsson E. Koper G. (2014). How to determine the core-shell nature in bimetallic catalyst particles? Catalysts 4, 375–396. 10.3390/catal4040375

[B28] Westsson E. Picken S. Koper G. (2019). The effect of lattice strain on catalytic activity. Chem. Commun. 55, 1338–1341. 10.1039/C8CC09063G 30638232

[B29] Zeng Z. Zhang T. Liu Y. Zhang W. Yin Z. Ji Z.. (2018). Magnetic field-enhanced 4-electron pathway for well-aligned Co3 O4 /electrospun carbon nanofibers in the oxygen reduction reaction. ChemSusChem 11, 580–588. 10.1002/cssc.201701947 29232499

[B30] Zhang J. (2008). PEM Fuel Cell Electrocatalysts and Catalyst Layers. London: Springer 10.1007/978-1-84800-936-3

[B31] Zheng Y. Jiao Y. Li L. H. Xing T. Chen Y. Jaroniec M.. (2014). Toward design of synergistically active carbon-based catalysts for electrocatalytic hydrogen evolution. ACS Nano 8, 5290–5296. 10.1021/nn501434a 24779586 PMC4046781

