# Supplementary material for: The Effect of Magnetic Field on Catalytic Properties in Core-Shell Type Particles
Source: Front Chem. 2020 Mar 12;8:163. doi: 10.3389/fchem.2020.00163 (PMC7082754; doi:10.3389/fchem.2020.00163)
Supplement: Supplementary file 1 [file Table_1.DOCX]

# Supporting information

| 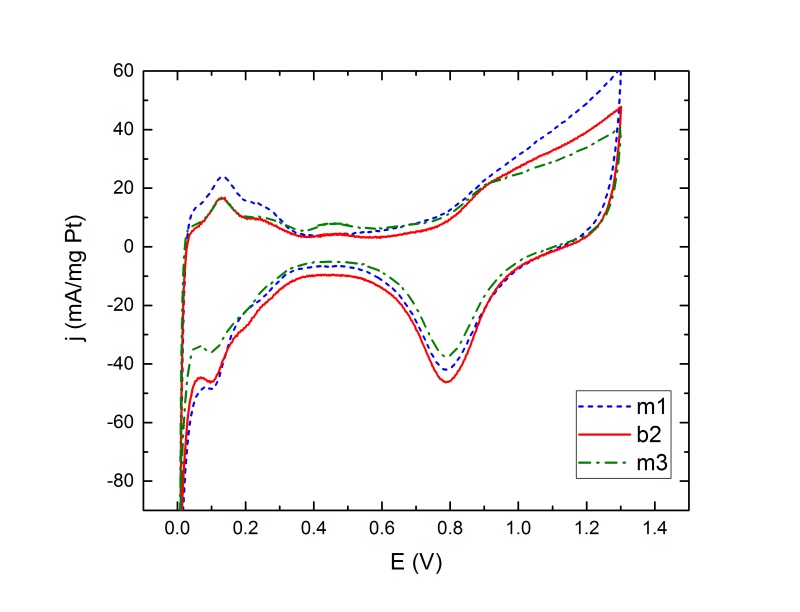  a | 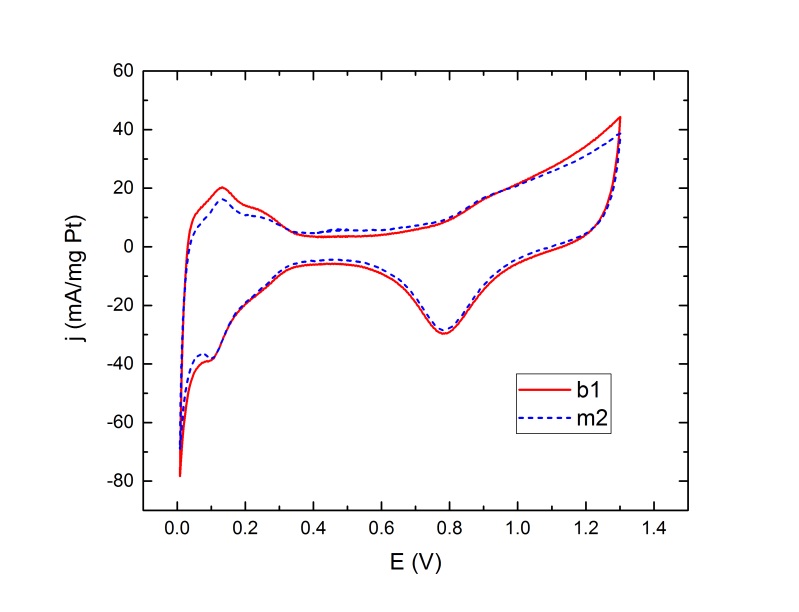  b |
| --- | --- |
| Fig S1 Cyclic voltammograms in Ar-saturated 0.1 M HClO_4_ electrolyte with Pt@Fe as catalyst. The two plots represent two different catalyst layer for which the catalyst layer in voltammogram a) was measured firstly in the magnetic configuration (“m1”), secondly non-magnetic configuration (“m2”) and thirdly with magnetic configuration again (“m3”). In voltammogram b) the layer was initially measured in non-magnetic configuration and then in magnetic configuration. Each set of measurements consisted of CV:s in Ar and a set of HV:s in O_2_. | |

| 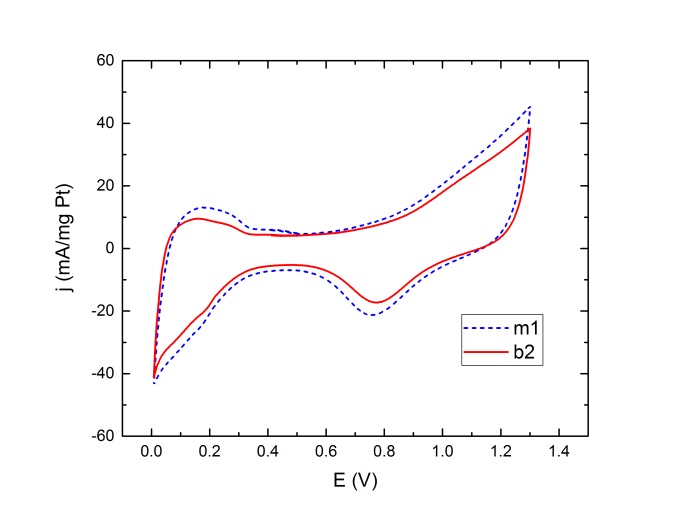  a | 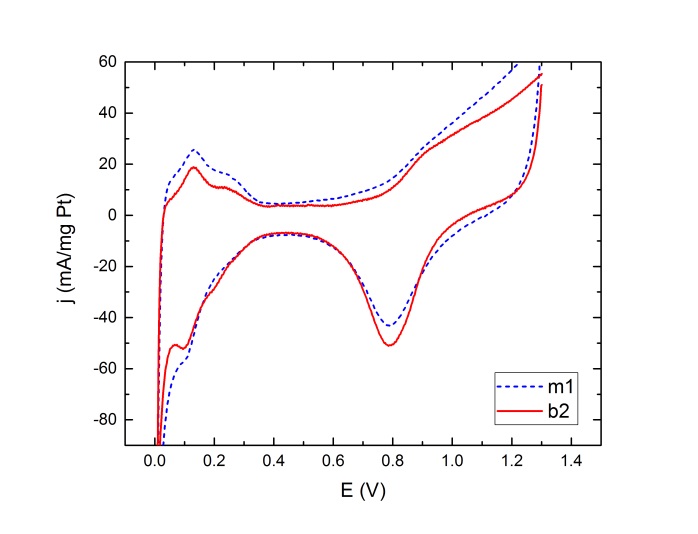  b |
| --- | --- |
| Fig S2 a) Pt@Cu at 50mV/s and b) Pt BME at 50 mV/s | |

| 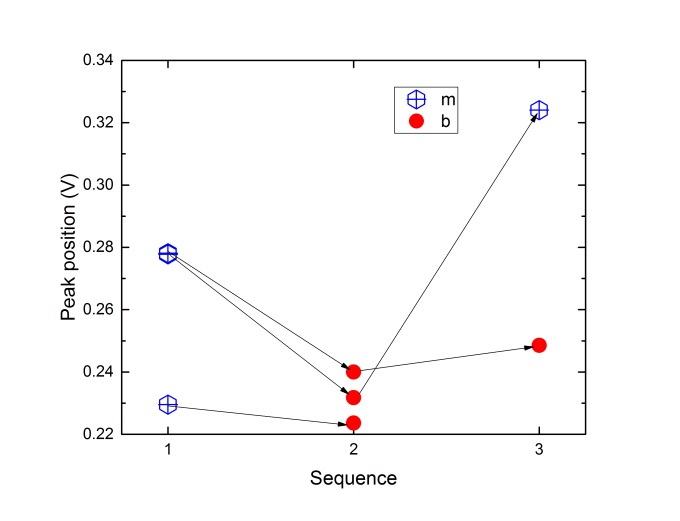  a | 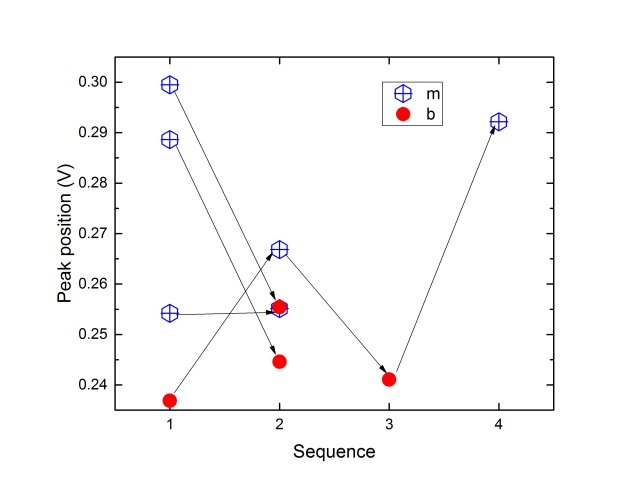  b |
| --- | --- |
| Figure S3 Positions for peak II as a function of measurement sequence at a) 50mV/s and b) 100mV/s scan speed | |

| 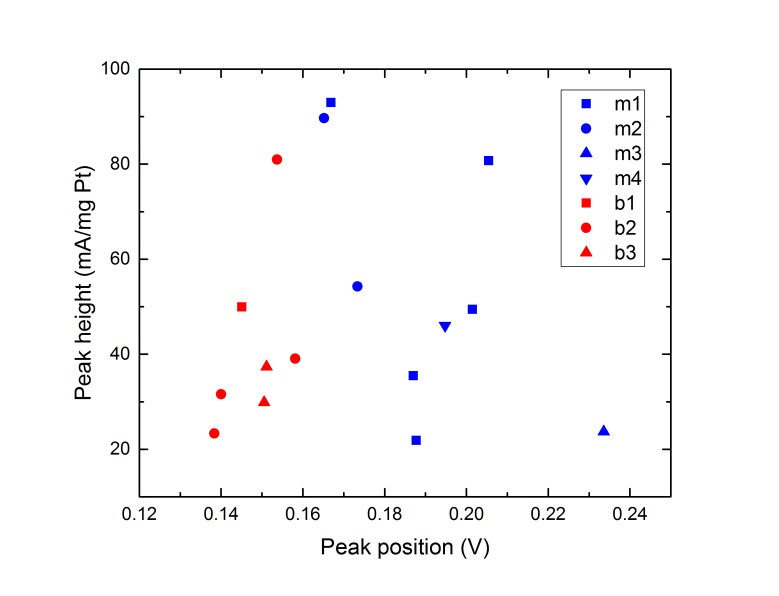 |
| --- |
| Figure S4 Peak height as a function of Peak position with legend indicating measurement sequence. |

| 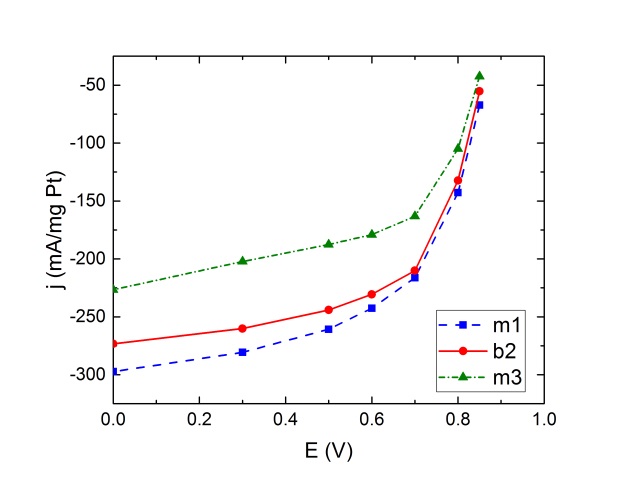  a | 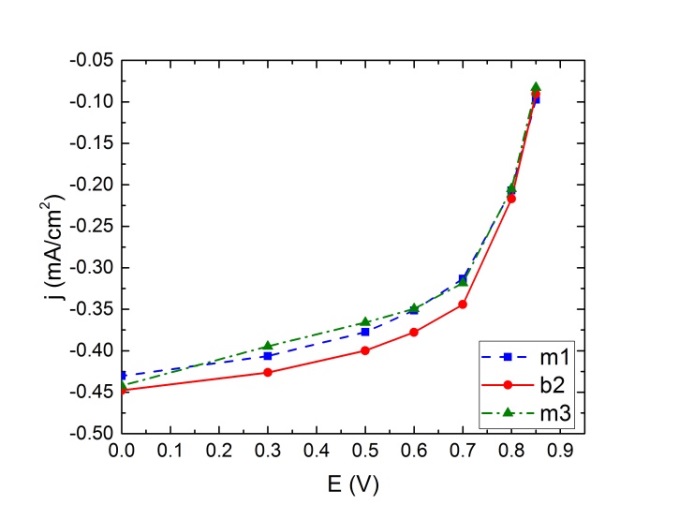  b |
| --- | --- |
| Fig S5 Hydrodynamic voltammograms for Pt@Fe illustrating the catalytic activity for oxygen reduction in the two different magnetic configurations, calculated both as a) mass activity and b) specific activity. | |


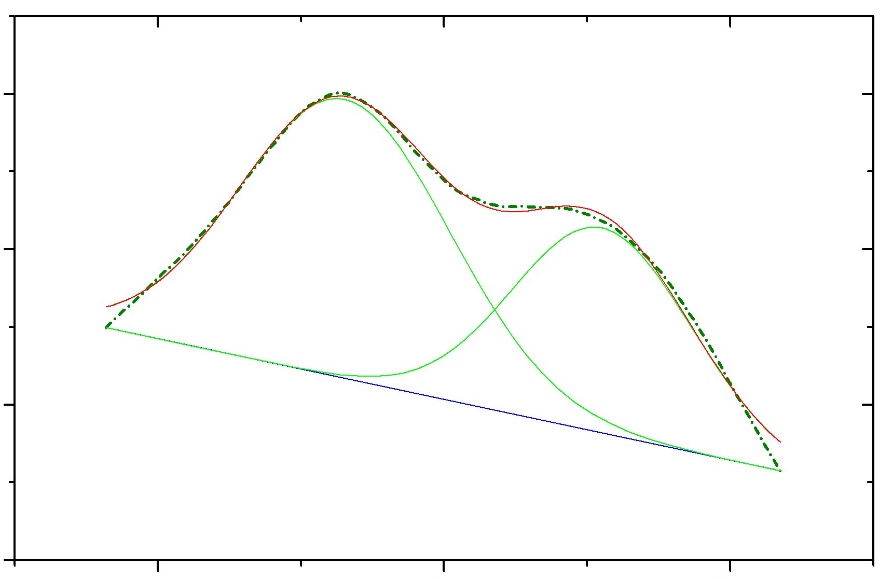


Fig S6 Peak fitting of hydrogen desorption peaks using two Gaussians.
